# Antimicrobial Resistance in *E. coli* and *Salmonella* spp. Across Romanian Animal–Food–Environment Interfaces: Current Evidence and One Health Knowledge Gaps

**DOI:** 10.3390/microorganisms14102144

**Published:** 2026-09-23

**Authors:** Iulia-Maria Bucur, Alex-Cristian Moza, Andrei Alexandru Ivan, Ingrid Dolores Moldovan, Alexandru Victor Udrea, Alexandra Adriana Grigoreanu, Alexandra Ban-Cucerzan, Ismail Amin, Emil Tirziu

**Affiliations:** 1 Animal Production and Veterinary Public Health Department, Faculty of Veterinary Medicine, University of Life Sciences “King Mihai I” from Timișoara, 300645 Timișoara, Romania; iulia.bucur@usvt.ro (I.-M.B.); ingrid.moldovan.iosud@usvt.ro (I.D.M.); alexandru.udrea.iosud@usvt.ro (A.V.U.); alexandra.grigoreanu@usvt.ro (A.A.G.); alexandracucerzan@usvt.ro (A.B.-C.); emiltirziu@usvt.ro (E.T.); 2 Microbiology and Parasitology Department, Faculty of Veterinary Medicine, Badr University in Cairo (BUC), Badr City 11829, Egypt; ismail.amin@buc.edu.eg

**Keywords:** multidrug resistance, antimicrobial resistance genes, food-producing animals, wastewater, genomic epidemiology

## Abstract

Antimicrobial resistance (AMR) is an important One Health challenge, yet evidence on resistant *Escherichia coli* and *Salmonella* spp. in Romania is dispersed across animal species, food matrices, environmental compartments, and surveillance systems. This comprehensive narrative review synthesizes and critically evaluates evidence across animal–food–environment interfaces and places it in the context of broader European AMR patterns. Particular attention is given to isolate origin, resistance phenotypes, multidrug resistance, major resistance determinants, molecular epidemiology, and the strength of evidence supporting cross-sector connections. Studies most frequently addressed poultry and swine, where resistance to ampicillin, fluoroquinolones, sulfonamides, and tetracyclines was repeatedly reported, together with extended-spectrum cephalosporin resistance, particularly in *E. coli*. Evidence from cattle, foods of animal origin, companion animals, wildlife, wastewater, and aquatic environments shows that resistant bacteria and antimicrobial resistance genes occur across multiple compartments. Substantial heterogeneity in sampling design, susceptibility testing, and molecular characterization limits direct comparison between studies, while epidemiologically linked investigations and whole-genome sequencing across connected reservoirs remain uncommon. Priorities include more standardized cross-sector surveillance, stronger environmental monitoring, expanded genomic epidemiology, improved data interoperability, and continued antimicrobial stewardship. By integrating fragmented national evidence, this review identifies both well-characterized sectors and interfaces where important evidence gaps remain.

## 1. Introduction

Antimicrobial resistance occurs when microorganisms no longer respond to antimicrobial agents, making infections more difficult or even impossible to treat. Although resistance may arise naturally, its selection and spread are accelerated by inappropriate antimicrobial use, inadequate infection prevention and sanitation, limited diagnostic capacity, and poor waste management. As a result, resistant infections often require longer treatment, last-resort drugs, additional testing, and prolonged hospitalization. In 2021, bacterial Antimicrobial resistance (AMR) was estimated to be associated with 4.71 million deaths worldwide, including approximately 1.14 million deaths directly attributable to resistance, with AMR-related mortality projected to rise further by 2050 [1].

The One Health approach recognizes the close interconnection between human, animal, and environmental health [2]. Resistant microorganisms, resistance genes, and antimicrobial residues can circulate across these sectors through food, direct contact, wastewater, manure, runoff, and wildlife. Therefore, AMR requires coordinated cross-sector surveillance rather than isolated investigation [2,3].

AMR has major clinical, economic, veterinary, and food-safety consequences. In humans, it limits treatment options, increases the risk of therapeutic failure, and raises healthcare costs. In livestock, resistant infections impair animal health, reduce treatment efficacy, and cause production losses, while resistant bacteria may reach consumers through contaminated food and food-processing environments. European surveillance continues to report substantial resistance in *Salmonella*, *Campylobacter*, and indicator *Escherichia coli*, highlighting the need for sustained control throughout the food chain [4].

Across Europe, AMR remains unevenly distributed, with higher resistance levels generally reported in southern, central, and eastern countries. European Antimicrobial Resistance Surveillance Network (EARS-Net) data for 2024 indicated persistently high resistance across the EU/EEA, including bloodstream infections caused by third-generation cephalosporin-resistant *Escherichia coli* and carbapenem-resistant *Klebsiella pneumoniae* [5,6]. Romania remains among the higher-burden countries, with elevated antimicrobial consumption and persistent limitations in surveillance, stewardship, infection prevention, and cross-sector coordination [5,7]. Although Romania contributes to EARS-Net and to harmonized EU monitoring of zoonotic and indicator bacteria, human, veterinary, food, and environmental data remain insufficiently integrated. Differences in sampling strategies, laboratory methods, and molecular characterization further limit cross-sector comparisons and source attribution, highlighting the need for interoperable databases, methodological harmonization, and systematic environmental surveillance within an integrated One Health framework [7,8,9].

Although antimicrobial resistance in Romania has been addressed in surveillance reports, individual research studies, and broader reviews, evidence concerning *E. coli* and *Salmonella* spp. remains dispersed across animal species, food matrices, environmental compartments, and geographically restricted investigations. This fragmentation makes it difficult to determine which resistance patterns are consistently supported across sectors, how Romanian findings relate to broader European trends, and where the most important evidence gaps remain. The present comprehensive narrative review therefore synthesizes and critically evaluates Romanian evidence on antimicrobial-resistant *E. coli* and *Salmonella* spp. across animal, food, and environmental interfaces. Particular attention is given to the epidemiological context of the isolates, resistance phenotypes and determinants, multidrug resistance, molecular and genomic evidence, and the strength of evidence supporting potential cross-sector connections. Romanian findings are also considered in relation to European data to distinguish broadly shared AMR patterns from observations that may warrant particular national attention. Finally, the review identifies methodological and surveillance gaps and outlines priorities for more integrated One Health investigation and AMR control in Romania.

## 2. Review Methodology

This study was conducted as a comprehensive narrative review of the available evidence on antimicrobial resistance in *Escherichia coli* and *Salmonella* spp. across Romanian animal, food, and environmental interfaces. Relevant literature was identified through structured searches of PubMed/MEDLINE, Scopus, and Web of Science, supplemented by Google Scholar and manual screening of reference lists. The final search was last updated on 15 September 2026. Search terms combined the target organisms with terms related to antimicrobial resistance, Romania, and relevant One Health compartments, including poultry, swine, cattle, meat, milk, dairy products, eggs, companion animals, wildlife, wastewater, surface water, soil, manure, and environmental sources.

Studies were included when they reported Romanian data on phenotypic antimicrobial resistance, multidrug resistance, resistance genes or mechanisms, molecular typing, or genomic characterization of *E. coli* or *Salmonella* spp. Studies involving food-producing animals, foods of animal origin, companion animals, wildlife, slaughter or processing environments, and environmental matrices were considered. Human studies were included when they contributed directly to the interpretation of animal–human, food–human, or environmental–human links. Official surveillance reports, policy documents, and regulatory sources from Romanian and European institutions were also considered for epidemiological and policy context but were distinguished from primary microbiological studies. Studies without Romanian data, without relevant AMR information, or without sufficient information on isolate origin were excluded. Studies without organism-specific AMR data were not included in the primary AMR evidence synthesis but were used selectively as contextual evidence when they informed food-chain hygiene, environmental dissemination pathways, antimicrobial exposure, or surveillance context.

For each eligible study, information on study year, geographical area, host or matrix, sampling source, number of samples or isolates, antimicrobial susceptibility-testing method and interpretive criteria, resistance frequencies, Multiple drug resistance(MDR)occurrence, resistance determinants, and molecular or genomic characterization was extracted whenever available. Because the included studies differed substantially in sampling design, populations, antimicrobial panels, laboratory methods, and interpretive standards, resistance frequencies were synthesized descriptively and were not statistically pooled. Wide ranges between studies were therefore interpreted as evidence of heterogeneity rather than as national prevalence estimates. Romanian findings were compared, where appropriate, with European surveillance data and peer-reviewed studies. The extracted information was used to compare and interpret findings across studies.

As this was a comprehensive narrative, no formal risk-of-bias assessment or meta-analysis was performed. Detection of similar resistance phenotypes, genes, serovars, or sequence types across different reservoirs was not considered evidence of direct transmission unless supported by epidemiologically linked sampling and sufficiently discriminatory molecular or genomic evidence.

## 3. The Romanian Context

European Centre for Disease Prevention and Control (ECDC) assessments have highlighted both high antibiotic consumption and extensive use of broad-spectrum agents [7]. Romanian hospital-based studies further indicate frequent use of cephalosporins, penicillins, fluoroquinolones, and other broad-spectrum antimicrobials [10,11]. Inappropriate antibiotic use is also influenced by public misconceptions, self-medication practices, premature treatment discontinuation, and pressure on physicians to prescribe antibiotics [12,13].

A recent survey among Romanian veterinarians working with food-producing animals identified stewardship challenges, including limited routine use of antimicrobial susceptibility testing, preference for broad-spectrum antimicrobials, reported prophylactic use, and frequent antimicrobial combinations [14]. These findings should be interpreted in the context of Regulation (EU) 2019/6, applicable since January 2022, under which routine antimicrobial use is prohibited and prophylactic antibiotic use is restricted to exceptional circumstances involving individual animals [15].

Romania participates in European AMR surveillance through national institutions and EU-coordinated monitoring networks. In the veterinary and food sectors, the National Sanitary Veterinary and Food Safety Authority (ANSVSA) implements standardized surveillance activities in accordance with Commission Implementing Decision (EU) 2020/1729. Surveillance includes *Salmonella* spp., commensal *E. coli*, and ESBL-/AmpC-/carbapenemase-producing *E. coli* from major food-producing animal populations and foods of animal origin [16]. At the human-health level, the National Institute of Public Health contributes data to the European Antimicrobial Resistance Surveillance Network (EARS-Net), coordinated by ECDC, which monitors resistance among invasive bacterial isolates across European countries [7]. Together, these systems provide important national data while enabling comparison with broader European trends.

Despite participation in harmonized European surveillance, important limitations remain. ECDC and WHO assessments have highlighted insufficient cross-sector coordination, limitations in surveillance and laboratory capacity, and the need to strengthen antimicrobial stewardship and AMR governance in Romania [7,17]. Recent assessments of the Romanian health system have similarly emphasized persistent challenges related to healthcare capacity, coordination, and the organization of public-health services [18]. Laboratory capacity and antimicrobial susceptibility testing practices also vary between institutions, while molecular surveillance and genomic characterization remain insufficiently integrated into routine surveillance. This is increasingly important as European AMR monitoring moves toward greater use of molecular methods and whole-genome sequencing for characterization and tracking of resistant pathogens [16,19]. Reporting limitations further affect the quality and consistency of national surveillance data. Persistent underreporting of healthcare-associated infections has been documented in Romania, while recent studies have identified manual reporting systems, workforce shortages, limited laboratory infrastructure, and concerns regarding institutional sanctions among the factors impairing accurate reporting [7,20,21].

These gaps remain relevant when Romania is considered against European antimicrobial-stewardship objectives. EU strategies emphasize the reduction of unnecessary and broad-spectrum antimicrobial exposure, standardized monitoring of antimicrobial consumption, coordinated AMR surveillance, and prudent antimicrobial use across human and veterinary medicine [22,23]. European analyses consistently place Romania among countries characterized by high antimicrobial consumption and resistance levels [24,25,26]. Strengthening antimicrobial stewardship, laboratory capacity and genomic surveillance, reporting quality, and cross-sector data integration therefore remains essential for developing a more effective One Health approach to AMR control in Romania.

Overall, the available Romanian evidence spans food-producing animals, foods of animal origin, companion animals, wildlife, human-associated investigations, and environmental matrices. The principal Romanian One Health compartments and the strength of evidence supporting connections between them are summarized in Figure 1.

## 4. AMR in Food-Producing Animals

Evidence from food-producing animals is concentrated in poultry and swine, with a smaller and more heterogeneous cattle literature. This uneven evidence distribution should also be viewed within the broader European context, where resistance in food-producing animals varies geographically and by host species, as demonstrated by recent predictive mapping of *E. coli*, *Salmonella*, and *Campylobacter* resistance across Europe [27]. Because sample origin, clinical status, and study design differ among investigations, findings are presented by species and interpreted within their specific epidemiological context.

### 4.1. Poultry

Poultry represents one of the best-characterized food-animal sectors for antimicrobial-resistant *E. coli*. Studies have examined commensal isolates from apparently healthy broilers and turkeys, cecal or fecal samples, slaughter-associated isolates, and production-chain samples [28,29,30,31,32]. Poultry-meat studies are considered separately in Section 4.4.1 to distinguish intestinal carriage from food contamination.

Among commensal *E. coli* recovered from slaughtered broilers, high resistance to fluoroquinolones, tetracyclines, ampicillin, and sulfonamides has been reported. In the national monitoring study of Romașcu et al. [32], resistance reached 89.5% to ciprofloxacin, 85.7% to nalidixic acid, 60.1% to tetracycline, 59.6% to ampicillin, and 59.4% to sulfamethoxazole, whereas resistance to third-generation cephalosporins was considerably less frequent. Other poultry investigations also reported frequent resistance to ampicillin, tetracycline, trimethoprim–sulfamethoxazole, and fluoroquinolones, although direct comparison of percentages is limited by differences in sample origin, antimicrobial panels, and interpretive criteria [28,29,30]. These values should therefore be interpreted as study-specific observations rather than estimates of national poultry prevalence.

Resistance to extended-spectrum cephalosporins is particularly relevant because several studies have demonstrated ESBL-associated and/or AmpC-associated *E. coli* in poultry. Rus et al. [30] reported presumptive ESBL/AmpC phenotypes in approximately 61% of broiler and 55% of turkey isolates in their study population, while molecular characterization identified β-lactam resistance determinants including *bla*TEM and AmpC-associated genes. Maciuca et al. [31] similarly demonstrated a high occurrence of extended-spectrum cephalosporin-resistant *E. coli* in broiler cecal samples, with CTX-M-type enzymes, including CTX-M-15, representing an important mechanism. Other investigations have identified additional resistance determinants involving tetracyclines, sulfonamides, aminoglycosides, and plasmid-mediated quinolone resistance [30,31,33,34]. These findings indicate that poultry-associated *E. coli* may carry multiple transferable resistance mechanisms, although the extent to which these determinants circulate clonally or through mobile genetic elements has rarely been assessed using high-resolution genomic approaches.

Multidrug resistance has also been documented, but reported proportions are not directly comparable across studies because MDR definitions, antimicrobial panels, and isolate-selection strategies differed. For example, a recent slaughter-chain investigation reported MDR among approximately one-third of its *E. coli* collection [28], whereas other studies focusing on resistant or ESBL/AmpC-enriched populations reported substantially greater resistance burdens [30,31]. Accordingly, the frequency of MDR should be considered in relation to the population from which isolates were selected rather than compared as a single range across studies.

For *Salmonella* spp., much of the available poultry-associated AMR information comes from carcasses, meat, and food-production samples rather than defined live-bird populations [35,36,37,38,39,40]. Those data are therefore discussed primarily in Section 4.4.1, where they can be interpreted at the relevant stage of the food chain.

Taken together, poultry studies show recurrent resistance to major antimicrobial classes and clinically relevant ESBL/AmpC mechanisms in *E. coli*. The overall resistance profile resembles EU monitoring patterns in poultry [4], but methodological differences preclude direct country-level prevalence comparisons. The principal unresolved issue is whether specific resistant lineages persist across bird–slaughter–food interfaces, which will require epidemiologically linked genomic sampling.

### 4.2. Swine

Studies document that antimicrobial-resistant *E. coli* and *Salmonella* spp. occur across several stages of the swine-production chain, including farm populations, clinically healthy pigs at slaughter, slaughterhouse environments, and pork products. However, these populations should not be considered epidemiologically equivalent. Farm-based studies have included growing pigs or piglets, whereas other investigations have examined cecal samples from apparently healthy slaughter pigs or isolates obtained during slaughter and processing [29,41,42,43,44,45,46]. Pork-derived isolates are discussed separately in Section 4.4.1 to avoid conflating intestinal carriage with food contamination.

For swine-associated *E. coli*, resistance to ampicillin, tetracyclines, fluoroquinolones, sulfonamides, and extended-spectrum cephalosporins has been repeatedly reported [29,42,43,44,45,46]. Bucur et al. [29], examining *E. coli* from clinically healthy slaughter pigs within a comparative animal–human study, reported resistance of 82.8% to ampicillin, 68.8% to trimethoprim–sulfamethoxazole, 65.6% to ciprofloxacin, 59.1% to ceftazidime, and 58.1% to cefotaxime among pig-derived isolates. These findings indicate a substantial resistance burden in that study population, but they should not be interpreted as national prevalence estimates.

Other investigations focused specifically on resistant or ESBL/AmpC-producing populations. Miliță et al. [42] reported high frequencies of resistance among ESBL/AmpC-producing commensal *E. coli* from slaughter pigs, including resistance to ampicillin, extended-spectrum cephalosporins, sulfonamides, tetracycline, trimethoprim, and ciprofloxacin. Similarly, Tipișcă et al. [45] identified ESBL-producing *E. coli* among cecal samples from healthy slaughter pigs in Botoșani County. Because these studies used selective approaches or resistance-enriched populations, their resistance percentages are not directly comparable with studies of unselected commensal isolates.

Farm-level investigations also demonstrate substantial resistance but involve different animal populations. Lungu et al. [44], studying *E. coli* from young pigs in western Romania, reported resistance to ampicillin in all tested isolates, together with frequent resistance to tetracycline and several β-lactams. Molecular analysis identified *amp*C, *bla*Z, *bla*TEM, and *tet*K, illustrating the coexistence of multiple resistance mechanisms. In another Western Romanian study, plasmid-mediated quinolone resistance determinants, particularly *qnr*B and *qnr*S, were detected in ciprofloxacin-resistant swine isolates [46]. These findings demonstrate that both β-lactam and quinolone resistance in swine may involve transferable resistance determinants.

Multidrug resistance has been reported across several swine-associated *E. coli* studies, although the reported proportions vary according to isolate selection, antimicrobial panel, and MDR definition. In the comparative study of Bucur et al. [29], pigs displayed a particularly high resistance burden relative to the other animal groups examined. Hulea et al. [43] also reported MDR among more than half of the swine-derived *E. coli* isolates analyzed. Such findings support continued attention to swine-associated *E. coli*, but cross-study numerical comparisons should remain cautious because the underlying populations and laboratory methodologies differ.

For *Salmonella*, evidence includes isolates from pigs, slaughter processes, and pork products [35,37,41,43,47,48]. Among swine- and slaughter-associated isolates, resistance to ampicillin, tetracyclines, sulfonamides, and quinolones has been reported, although resistance frequencies varied considerably between studies [41,43,47,48]. Morar et al. [41], examining isolates from an industrial pig slaughter and processing system, reported resistance to tetracycline in 61.5%, ampicillin in 50%, and trimethoprim–sulfamethoxazole in 34.6% of isolates, while resistance to ciprofloxacin was low and no resistance to cefotaxime or ceftazidime was detected. In contrast, studies involving pork from traditional slaughter systems reported much higher MDR frequencies [48]. These differences likely reflect variation in sample source, production setting, serovar distribution, antimicrobial exposure, and testing methodology rather than a uniform national pattern.

Molecular characterization of swine-derived *Salmonella* is less developed than that of *E. coli*. Romanian studies have frequently reported serovars such as *S. Typhimurium*, *S. Derby*, *S. Infantis*, and *S. Rissen* [35,41,47,48], but detailed investigation of β-lactamase genes, plasmid-mediated quinolone resistance, mobile genetic elements, and genomic relatedness remains comparatively limited in swine-associated *Salmonella*. This limits assessment of whether similar resistance profiles arise through clonal dissemination or independent acquisition of mobile resistance determinants. ESBL/AmpC-producing *E. coli* are well documented in European pig populations, often together with resistance to fluoroquinolones, tetracyclines, and sulfonamides [4,49,50,51,52,53,54]. European surveillance also identifies *S. Typhimurium*, *S. Derby*, *S. Rissen*, and *S. Infantis* among relevant swine-associated *Salmonella* serovars, with frequent resistance to ampicillin, sulfonamides, and tetracyclines, whereas resistance to third-generation cephalosporins and carbapenems generally remains less common [4,55,56,57].

Swine studies demonstrate recurrent resistance to several major antimicrobial classes in both *E. coli* and *Salmonella* spp., with particularly strong molecular evidence for ESBL/AmpC and plasmid-mediated quinolone resistance in *E. coli*. However, available studies encompass different populations, including farm animals, clinically healthy slaughter pigs, slaughter environments, and pork products, which limits direct comparison of resistance frequencies. Molecular and genomic characterization of swine-associated *Salmonella* remains comparatively sparse, and epidemiologically linked studies following resistant strains across farm, slaughter, food, and environmental compartments are still needed.

### 4.3. Cattle

Evidence on antimicrobial resistance in cattle is more heterogeneous than that available for poultry and swine because the published studies encompass different epidemiological contexts, including pathogenic slaughter-associated isolates, bovine mastitis, postpartum uterine infections, and mixed young-animal datasets that include calves. These sources should be considered separately because resistance patterns in commensal or slaughter-associated isolates may differ substantially from those recovered from clinical disease. Studies of raw milk and dairy products are discussed separately in Section 4.4.2 because they represent food-chain rather than animal-clinical matrices.

Among cattle-associated *E. coli*, resistance to β-lactams, fluoroquinolones, tetracyclines, sulfonamides, and aminoglycosides has been reported, although the magnitude of resistance varies according to population, clinical status, and study design [58,59,60,61].

Tăbăran et al. [58] examined pathogenic *E. coli* recovered from cattle at slaughter and reported multidrug resistance in more than two-thirds of the Romanian pathogenic isolates included in the analysis. Molecular characterization demonstrated several transferable resistance determinants, including genes associated with tetracycline, sulfonamide, aminoglycoside, and trimethoprim resistance, together with class 1 integrons. These findings indicate that bovine *E. coli* may carry combinations of resistance determinants with potential for horizontal dissemination, although the study population consisted of selected pathogenic isolates rather than an unselected cattle population.

Clinical dairy-cattle studies provide a different perspective. In cows with postpartum endometritis, *E. coli* was one of the predominant Gram-negative pathogens identified, and resistance was particularly noted to tetracycline and ampicillin, whereas susceptibility to imipenem remained preserved [59]. In bovine mastitis, *E. coli* isolates also showed resistance to several antimicrobial classes, although the number of isolates was relatively small and the resistance pattern differed from that reported in slaughter-associated populations [60]. These differences reinforce the need to distinguish clinical isolates from commensal or slaughter-derived *E. coli* when interpreting cattle-associated AMR.

Historical data also suggest that resistance patterns have changed over time. Chirilă et al. [61] examined Shiga toxin-producing *E. coli* recovered from young animals over several decades and reported an increase in multidrug resistance in more recent isolates. The study also identified *tet*, *sul*, aminoglycoside- and trimethoprim-resistance genes, as well as class 1 integrons. However, this dataset included calves together with piglets and foals and therefore should not be interpreted as cattle-specific prevalence evidence. Its main relevance to the present review is the demonstration that resistance determinants and MDR have circulated among pathogenic *E. coli* from young livestock in Romania over an extended period.

The molecular evidence available for cattle-associated *E. coli* is therefore more informative regarding the types of resistance mechanisms present than their national prevalence. Genes encoding tetracycline resistance, sulfonamide resistance, aminoglycoside resistance, trimethoprim resistance, and integron-associated determinants have been reported [58,61]. Nevertheless, high-resolution genomic studies linking cattle isolates to milk, food products, humans, or environmental sources remain poorly represented. Consequently, the available evidence does not allow robust assessment of whether resistant bovine lineages persist along the dairy or beef production chain or whether similar resistance determinants arise independently in different compartments.

In contrast to *E. coli*, cattle-associated *Salmonella* AMR is markedly underrepresented in the Romanian literature. No eligible primary studies were identified in the evidence evaluated for this review that provided antimicrobial susceptibility data for *Salmonella* isolated from cattle, calves, bovine feces, bovine carcasses, raw beef, or cattle-associated farm environments. This contrasts with the substantially larger Romanian evidence bases for poultry- and swine-associated *Salmonella* and limits comparison of the relative contribution of cattle to the national food-animal AMR landscape.

European surveillance likewise documents resistant and ESBL/AmpC-producing *E. coli* in cattle, although resistance patterns vary with production system, age group, antimicrobial exposure, and sampling framework [4]. In the evidence evaluated here, the more important limitation is the comparatively fragmented cattle evidence base, particularly for standardized monitoring, genomic characterization, and cattle-associated *Salmonella*.

Across food-producing species, resistant *E. coli* and/or *Salmonella* spp. have also been detected in apparently healthy or slaughter-age animals. This asymptomatic carriage is especially relevant for commensal *E. coli*, which may harbor transferable resistance determinants without clinical disease. Healthy populations should therefore be included in AMR surveillance, while recognizing that carriage alone does not demonstrate onward transmission to food, humans, or the environment [29,32,42,45].

### 4.4. Foods of Animal Origin

#### 4.4.1. Meat and Meat Products

Meat and meat-product studies include poultry meat, pork, processed products, and slaughter- or retail-associated matrices. Because sampling stage, product type, and susceptibility methods vary, the reported frequencies are interpreted as study-specific observations rather than pooled estimates.

For *E. coli*, poultry meat has been investigated more extensively than pork. Brătfelan et al. [33] recovered *E. coli* from chicken meat and reported frequent resistance to ampicillin and tetracycline, together with resistance to sulfonamides, chloramphenicol, quinolones, and extended-spectrum cephalosporins. Molecular analysis identified several resistance determinants, including *bla*TEM, CTX-M-associated β-lactamase genes, *tet*, *sul*, aminoglycoside-resistance, and plasmid-mediated quinolone-resistance determinants. Other Romanian poultry-meat investigations similarly identified resistant *E. coli* and genes associated with β-lactam, tetracycline, and sulfonamide resistance [34,39]. These studies indicate that poultry meat can contain *E. coli* carrying multiple resistance determinants, but they do not establish whether resistant strains originated from intestinal carriage, slaughter contamination, processing, or subsequent handling.

Resistance has also been documented in *E. coli* recovered from other meat-associated matrices. Mateescu et al. [62] identified resistant *Enterobacteriaceae*, including *E. coli*, in foods marketed in Bucharest and reported resistance to ampicillin, tetracycline, and several β-lactams, together with ESBL-associated determinants. Gașpar et al. [63] additionally recovered resistant *E. coli* from absorbent pads used in meat packaging. The study included an isolated phenotypic observation of meropenem resistance; however, the available evidence does not demonstrate confirmatory carbapenemase testing or molecular characterization of the mechanism involved. This finding should therefore be interpreted cautiously and does not provide evidence of established carbapenem-resistant *E. coli* circulation in Romanian retail meat.

For *Salmonella*, poultry meat and pork represent the best-documented food matrices. Poultry-associated studies have frequently identified *S. Enteritidis*, *S. Infantis*, and *S. Typhimurium*, together with occasional detection of other serovars [35,36,37,38,39,40]. Resistance to tetracycline, quinolones, sulfonamides, and ampicillin has been repeatedly reported, although the magnitude varied considerably among studies. In some poultry-meat investigations, resistance to nalidixic acid and ciprofloxacin was particularly frequent [36,38]. Molecular studies further identified β-lactamase, quinolone-resistance, tetracycline-resistance, and sulfonamide-resistance determinants in selected isolates [38,40].

Pork-associated *Salmonella* studies similarly reported *S. Typhimurium*, *S. Derby*, *S. Rissen*, and *S. Infantis* among the predominant serovars [35,41,47,48]. Morar et al. [41], examining isolates recovered during an industrial pig slaughter and processing system, reported resistance particularly to tetracycline and ampicillin, whereas resistance to fluoroquinolones and extended-spectrum cephalosporins was comparatively uncommon. By contrast, Tăbăran et al. [48] reported a very high frequency of MDR among *Salmonella* isolates recovered from pork produced in traditional slaughter systems. These differences illustrate the influence of production context, serovar composition, isolate selection, and susceptibility-testing methodology on apparent resistance frequencies.

MDR has been reported in both poultry- and pork-derived isolates, but direct comparison between studies remains difficult. Some investigations evaluated all recovered isolates, whereas others focused on selected resistant populations or particular serovars. Antimicrobial panels and MDR definitions also differed. For this reason, individual MDR percentages are more informative when interpreted together with the study population and analytical methods rather than when presented as a single national range.

Taken together, meat studies confirm resistant *E. coli* and *Salmonella* spp. most consistently in poultry meat and pork. European data likewise identify these matrices as relevant for ESBL/AmpC-producing *E. coli* and MDR *Salmonella*, including *S. Infantis* [4,55,64,65,66,67,68,69,70,71,72]. Most available investigations, however, are cross-sectional and cannot establish persistence of the same resistant clones from animals through slaughter, processing, and retail.

#### 4.4.2. Milk and Dairy Products

Evidence from milk and dairy products is smaller than that from meat but includes raw cow milk, raw ovine milk, and raw-milk cheeses. These matrices may reflect animal infection or carriage as well as contamination during milking, processing, handling, or retail; differences in production systems and testing methods therefore require study-specific interpretation.

In raw cow milk, Drugea et al. [73] investigated a large collection of samples obtained from dairy farms and reported *E. coli* among the bacterial pathogens recovered. Resistance was particularly frequent to penicillin–novobiocin and streptomycin, and temporal variation in susceptibility to several antimicrobials was observed during the study period. Multidrug resistance was identified in 27.5% of the *E. coli* isolates. Because this investigation focused on raw milk rather than clinically characterized individual animals, the findings are best interpreted as evidence of resistance at the milk-production interface rather than as estimates of AMR among bovine clinical isolates.

Additional evidence is provided by Roșu et al. [74], who examined *E. coli* recovered from raw ovine bulk-tank milk in the Banat region. Among 48 isolates obtained from 72 milk samples, resistance was most frequent to ampicillin (45.8%), followed by gentamicin (20.8%), ticarcillin–clavulanate (18.8%), and cephalexin (18.8%), while no resistance to imipenem was detected. Five isolates were multidrug-resistant (10.4%), and *stx*2 was identified in 9/48 isolates (18.8%). These findings extend the dairy AMR evidence beyond cattle and indicate that small-ruminant milk should also be considered in food-chain surveillance.

Resistance has also been documented in dairy products. Imre et al. [75] investigated traditionally produced raw-milk cheese commercialized in the Banat region and recovered 43 *E. coli* isolates from 53 products. Resistance was reported most frequently to ampicillin, followed by norfloxacin and amoxicillin–clavulanate, while resistance to extended-spectrum cephalosporins was less common. Ten isolates were multidrug-resistant. These findings confirm that resistant *E. coli* may be detected at the final-product stage, although the study design does not establish whether contamination originated from raw milk, the processing environment, handlers, or post-production handling.

Mateescu et al. [62] additionally identified antimicrobial-resistant Enterobacteriaceae, including *E. coli*, in a heterogeneous group of foods marketed in Bucharest that included milk, cheese, and yogurt. Resistance to several β-lactams and tetracycline was documented, together with β-lactamase- and tetracycline-resistance determinants. However, the small numbers of isolates from individual dairy-product categories limit matrix-specific interpretation, and these findings are more appropriately considered evidence of occurrence than prevalence estimates.

Other investigations provide complementary information on dairy-chain microbiological quality and safety without organism-specific AMR testing. Suciu et al. [76] detected *E. coli* in raw milk from small-scale backyard cattle farms in western Romania and investigated pathogenicity-associated markers, while additional studies assessed raw milk sold through vending machines in Timișoara and Arad, raw milk from a processing plant in Cluj County, and traditionally produced cheeses marketed in Bihor County [77,78,79,80]. Together, these studies provide broader context on hygiene, microbial contamination, and antimicrobial residues along the dairy chain.

The available dairy literature is dominated by *E. coli*, whereas primary evidence for antimicrobial-resistant *Salmonella* in raw milk and dairy products remains much more limited. This imbalance may reflect study design and organism-specific research priorities rather than absence of *Salmonella* from the dairy chain.

At the European level, resistant *E. coli*, including ESBL-producing isolates, have also been reported from raw milk and dairy-production environments, although occurrence varies according to production system, hygiene conditions, antimicrobial exposure, and analytical approach [81,82]. The available evidence therefore supports milk and dairy products as potential vehicles for resistant *Enterobacterales*, but does not allow their relative contribution to AMR dissemination to be compared directly with that of meat-associated food chains.

Overall, resistant and multidrug-resistant *E. coli* have been documented in raw cow milk, raw ovine milk, and raw-milk dairy products, while genomic linkage between animals, milk, processing environments, and final products remains largely unexplored. Coordinated farm-to-product sampling combined with molecular or genomic comparison would help determine whether resistant strains persist along the dairy-production chain.

#### 4.4.3. Eggs

Evidence on antimicrobial-resistant *Salmonella* in eggs is limited. Tîrziu et al. [47] included 48 shell-egg samples in a broader food study and recovered three *Salmonella* isolates: two *S. Enteritidis* and one *S. Infantis*. Individual egg-derived isolates were resistant to between two and seven of the tested antimicrobials. The small isolate number precludes a prevalence estimate, but it confirms that resistant *Salmonella* can occur in this matrix.

*S. Enteritidis* is well recognized in egg-associated epidemiology, while *S. Infantis* is increasingly important in European poultry AMR surveillance [4].

The available dataset is too small for robust comparison with poultry meat or pork and contains no epidemiologically linked molecular or genomic investigation connecting laying hens, farm environments, eggs, and human isolates. Larger studies using standardized susceptibility testing and molecular characterization are therefore needed.

## 5. Companion Animals and Human–Animal Interfaces

Companion animals represent a distinct One Health interface because they share households and other close-contact environments with humans and may receive antimicrobial treatment outside food-animal production systems. Romanian studies have documented antimicrobial-resistant *E. coli* and *Salmonella* spp. in dogs, cats, reptiles, pet birds, and other companion species, although study designs range from population-based surveys to individual clinical case reports [83,84,85,86,87,88,89,90,91].

Among dogs, resistant *E. coli* has been identified in both healthy and clinically affected animals. Cozma et al. [83] investigated healthy dogs and identified extended-spectrum cephalosporin-resistant Enterobacterales, predominantly *E. coli*, including ESBL-producing isolates carrying CTX-M-, TEM-, SHV-, and plasmid-mediated quinolone-resistance determinants. These findings demonstrate asymptomatic carriage of clinically relevant resistance mechanisms in companion animals and indicate that healthy dogs may contribute to the maintenance of resistant *Enterobacterales* within domestic environments.

Clinical canine populations show a different epidemiological context. Popa et al. [84] recovered *E. coli* from dogs with otitis externa and reported resistance particularly to aminoglycosides, together with multidrug-resistant and phenotypically ESBL-producing isolates. Because these isolates originated from clinical infections rather than asymptomatic carriage, their resistance profiles should not be compared directly with those recovered from healthy dogs.

The strongest Romanian evidence for human–companion-animal genomic relatedness comes from the study of Cozma et al. [85], which investigated extended-spectrum cephalosporin-resistant *Enterobacterales* in dogs and humans sharing households or animal-shelter environments. Whole-genome sequencing and molecular typing identified closely related *E. coli* isolates in dogs and humans occupying the same environment, including shared sequence types and resistance profiles. This study provides stronger evidence of cross-host circulation than studies based solely on similar resistance phenotypes or individual resistance genes. Nevertheless, genomic relatedness alone does not establish the direction of transmission, which could involve dog-to-human, human-to-dog, or acquisition from a shared environmental source.

Romanian data also demonstrate resistant *Salmonella* in companion animals. Dégi et al. [86] identified *Salmonella* in 16 of 85 client-owned urban cats, with *S. Enteritidis*, *S. Typhimurium*, and *S. Kentucky* among the recovered serovars. All isolates were resistant to at least three antimicrobials, and resistance to ampicillin, trimethoprim–sulfamethoxazole, aminoglycosides, and extended-spectrum cephalosporins was reported. These findings demonstrate that companion cats may carry MDR *Salmonella*, although the study did not establish epidemiological links with food-animal or human isolates.

A particularly relevant cross-source investigation was reported by Colobatiu et al. [40]. The study included *Salmonella* isolates from humans, food, and one dog and identified plasmid-mediated quinolone-resistance and β-lactamase determinants. Pulsed-Field Gel Electrophoresis(PFGE)showed that the canine *S. Infantis* isolate shared a closely related or indistinguishable profile with isolates recovered from chicken meat and a human source. This observation supports cross-source molecular similarity but, because sampling was not prospectively epidemiologically linked and PFGE provides lower resolution than WGS, it should not be interpreted as proof of direct transmission.

Pet reptiles represent another companion-animal interface. Romanian investigations identified resistant *Salmonella* and *E. coli* in reptiles maintained as companion animals [87,88]. In the Timișoara metropolitan area, Dégi et al. [88] reported MDR in 23 of 29 reptile-level *Salmonella* isolates subjected to resistance analysis. Given the known potential for direct contact between reptiles, their environments, and household members, such findings support consideration of reptiles within companion-animal AMR surveillance, although human transmission was not investigated directly.

Evidence from pet birds and less frequently studied companion species further broadens the range of potential reservoirs. Resistant *E. coli* has been reported in ornamental birds [89], while recent Romanian case reports described MDR *E. coli* causing systemic disease in a domestic rabbit [90] and a guinea pig [91]. Both case reports identified combinations of resistance determinants including *bla*CTX-M, *bla*TEM, *tet*A, and *sul*1. These studies demonstrate the occurrence of clinically relevant resistance mechanisms in uncommon companion species, but, as individual case reports, they cannot provide prevalence estimates or population-level risk assessments.

Taken together, the companion-animal evidence differs substantially in strength. Population studies demonstrate carriage or infection with resistant bacteria, molecular investigations identify clinically relevant antibiotic resistance genes(ARGs), and one whole-genome sequencing (WGS)-based dog–human study [85] provides direct evidence of close genomic relatedness between hosts sharing the same environment. In contrast, similar serovars or resistance genes identified in unrelated hosts should be regarded as evidence of overlap rather than transmission. The relevance of this interface is also recognized more broadly in Europe, where antimicrobial use and resistant bacteria in companion animals are increasingly considered within One Health AMR surveillance and stewardship frameworks [92].

Companion animals therefore warrant inclusion in One Health AMR surveillance, particularly in settings involving close human contact, while broader linked studies are needed to determine the frequency and direction of cross-host circulation.

## 6. Wildlife as Sentinels and Potential Reservoirs of AMR

Wildlife represents an additional component of the Romanian One Health AMR landscape because free-ranging animals may be exposed to resistant bacteria through agricultural environments, surface waters, waste, livestock contact, or other anthropogenic sources while generally receiving little or no direct antimicrobial treatment. Romanian evidence remains smaller than that available for livestock or companion animals, but studies in wild ungulates and red foxes demonstrate the occurrence of antimicrobial-resistant *E. coli* and *Salmonella* spp. in free-ranging populations [93,94,95].

In fallow deer from western Romania, Tîrziu et al. [93] investigated bacteria recovered from nasal and rectal samples and identified phenotypic resistance among *E. coli* isolates, including resistance to several β-lactam antimicrobials. Because the number of *E. coli* isolates subjected to antimicrobial susceptibility testing was relatively small, these findings should be interpreted as evidence of occurrence rather than as estimates of resistance prevalence in Romanian fallow-deer populations.

Additional molecular evidence has recently been obtained from fallow deer in western Romania. In a subsequent study, resistant *E. coli* isolates were investigated for β-lactam-resistance determinants, with *bla*TEM and *bla*SHV detected among selected isolates. Although based on a limited isolate collection, these findings extend the wildlife evidence beyond phenotypic resistance and demonstrate the presence of clinically relevant resistance genes in wildlife-associated *E. coli* [96].

Red foxes provide evidence from a different ecological niche. Moza et al. [94] examined bacterial isolates recovered from red foxes across hunting areas in western Romania and identified resistant *E. coli* as well as *S. enterica*. Resistance profiles varied geographically, and multidrug resistance was observed in selected isolates. Because red foxes occupy peri-urban and peri-rural environments and may interact indirectly with livestock areas, waste, and human-modified habitats, they may be useful sentinels of environmental AMR exposure. However, the available data do not establish the source from which resistant bacteria were acquired.

More recently, Pătrînjan et al. [95] investigated *E. coli* recovered from mesenteric lymph nodes of free-ranging wild boars and cervids in western Romania. Among 75 *E. coli* isolates, resistance to at least one antimicrobial was reported in 25.3%, while 20.0% were classified as multidrug-resistant. Ampicillin and cephalexin were among the antimicrobials for which resistance was most frequently observed. The recovery of resistant *E. coli* from mesenteric lymph nodes is particularly relevant because it demonstrates bacterial carriage beyond superficial environmental contamination, although the study did not investigate genomic relationships with livestock, food, or human isolates.

The Romanian wildlife evidence should therefore be interpreted primarily in terms of occurrence and environmental exposure, rather than demonstrated transmission. Detection of resistance phenotypes or resistance genes in wildlife may reflect exposure to anthropogenic AMR sources, persistence of resistant bacteria within wildlife populations, or acquisition from shared environmental reservoirs. Without contemporaneous sampling of wildlife, livestock, water, soil, food, and humans, it is not possible to distinguish among these possibilities.

At the European level, antimicrobial-resistant *Enterobacterales* have also been documented in free-ranging wildlife, supporting the role of wild animals as sentinels of environmental AMR exposure [97]. Romanian observations are therefore compatible with the broader concept of wildlife as both sentinels of environmental contamination and potential secondary reservoirs of resistant bacteria. Nevertheless, the limited number of Romanian studies and the predominance of phenotypic rather than genomic characterization currently prevent robust assessment of wildlife contribution to AMR dissemination.

Romanian studies confirm the occurrence of antimicrobial-resistant and multidrug-resistant *E. coli* and *Salmonella* spp. in several free-ranging wildlife species, with recent evidence also identifying β-lactam-resistance genes in fallow-deer *E. coli*. These findings support the value of wildlife for AMR surveillance, particularly as sentinels of environmental exposure. However, no epidemiologically linked genomic studies currently demonstrate transmission between Romanian wildlife, livestock, food, environmental, or human compartments; future investigations should therefore combine wildlife sampling with contemporaneous sampling of adjacent agricultural and environmental sources.

## 7. Environmental Dimensions of AMR

Environmental compartments represent an important component of the Romanian AMR landscape because wastewater, agricultural effluents, surface waters, and other environmental matrices may receive resistant bacteria, ARGs, and antimicrobial residues from both human and animal sources [98]. However, the strength of the Romanian evidence varies considerably among environmental pathways. Direct organism-specific evidence is strongest for wastewater and aquatic environments, whereas landfill and bioaerosol studies mainly provide contextual evidence of microbial dissemination rather than direct demonstration of antimicrobial-resistant *E. coli* or *Salmonella* spp.

### 7.1. Urban, Hospital, and Aquatic Wastewater

Hospital and municipal wastewater represent the best-characterized environmental AMR compartments in Romania. Szekeres et al. [99] investigated Romanian hospital wastewater and demonstrated the persistence of antimicrobial residues and multiple resistance-associated determinants despite wastewater treatment. Genes including *sul*1 and *qac*EΔ1 remained detectable after treatment, while the bacterial community continued to contain members of the Enterobacteriaceae. These findings provide direct evidence that healthcare wastewater can introduce antimicrobial residues and ARGs into downstream treatment systems and receiving environments, although the study was not designed specifically to quantify resistant *E. coli* or *Salmonella* populations.

Polianciuc et al. [100] similarly examined influent and effluent from Romanian municipal wastewater-treatment plants and identified both antimicrobial residues and phenotypically resistant bacteria. Resistance was observed particularly to amoxicillin and clarithromycin, with additional resistance or reduced susceptibility to fluoroquinolones in selected isolates. Because the bacterial isolates were not characterized sufficiently to provide organism-specific estimates for the two target pathogens, this study is best interpreted as direct evidence of environmental AMR rather than as *E. coli*- or *Salmonella*-specific surveillance.

More detailed organism-level evidence is provided by Farkas et al. [101], who investigated *Enterobacterales* from surface water, groundwater, municipal wastewater, hospital effluent, and farm effluent in north-western Romania. Among 678 *Enterobacterales* isolates, resistant *E. coli* strains were identified together with resistance determinants including *blaTEM-1*, *tetA*, and *intI1*. Approximately one-third of the complete *Enterobacterales* collection was multidrug-resistant, while carbapenem resistance was uncommon. ERIC-PCR also identified identical or closely related fingerprints among isolates recovered from some distinct aquatic matrices, indicating possible environmental connectivity. Nevertheless, this level of molecular similarity is insufficient to establish a specific direction of dissemination or source attribution.

Taken together, these studies demonstrate that Romanian urban and healthcare wastewater systems contain antimicrobial residues, resistant bacteria, and transferable resistance determinants and that conventional treatment does not necessarily eliminate them completely. However, the available studies use different analytical approaches—ranging from residue and ARG quantification to culture-based susceptibility testing—and should therefore not be combined into a single quantitative estimate of environmental resistance.

Wastewater from pharmaceutical manufacturing is recognized internationally as a potential AMR-selection environment, and this pathway is discussed in broader environmental reviews [98]. However, no Romanian primary study specifically characterizing antimicrobial-resistant *E. coli* or *Salmonella* from pharmaceutical-production wastewater was represented in the evidence evaluated for this review. It should therefore be regarded as a relevant surveillance gap rather than as a demonstrated Romanian source.

### 7.2. Livestock-Associated Effluents, Manure, Soil, and Agricultural Pathways

Livestock production provides a separate potential route by which resistant bacteria and ARGs may reach the environment through feces, manure, farm wastewater, runoff, and agricultural application of animal waste. Compared with hospital and municipal wastewater, Romanian primary evidence specifically linking resistant *E. coli* or *Salmonella* from livestock to surrounding soils or waters remains relatively limited.

Farkas et al. [101] provide the clearest direct evidence because farm effluent was included alongside municipal, hospital, groundwater, and surface-water matrices. Resistant *Enterobacterales*, including *E. coli*, were detected in livestock-associated effluent, showing that farm discharges may contain organisms with clinically relevant resistance phenotypes and determinants. Nevertheless, because animal isolates from the corresponding farms were not sampled and genomically compared with environmental isolates, the study cannot determine whether particular resistant strains originated directly from livestock.

The broader pathway from manure to agricultural soil is biologically plausible and well established in the international literature, while Romanian reviews have highlighted the potential contribution of manure to environmental ARG dissemination [102]. However, because [102] is a review rather than a Romanian primary AMR study, it should be used as contextual support rather than counted as direct national evidence. Similarly, the current Romanian literature does not yet provide a well-developed series of paired animal–manure–soil–water studies capable of quantifying persistence or transmission of individual *E. coli* or *Salmonella* lineages.

This distinction is important when interpreting the One Health significance of agricultural environments. The presence of the same resistance gene in livestock and environmental samples may result from movement of resistant bacteria, horizontal transfer of mobile genetic elements, or independent selection under similar antimicrobial pressures. Demonstrating movement between these compartments would require coordinated sampling and high-resolution genomic analysis.

### 7.3. Landfill and Airborne Pathways

Romanian landfill and livestock-bioaerosol investigations provide additional information on the potential for environmental microbial dissemination, although their relevance to organism-specific AMR is indirect. Pop et al. [103] documented substantial microbial contamination in and around a Romanian municipal solid-waste landfill, supporting the potential for airborne dispersal of Gram-negative and other bacteria from waste-management environments. However, the study did not specifically characterize antimicrobial-resistant *E. coli* or *Salmonella* and should not be interpreted as direct AMR evidence for these organisms.

Similarly, studies conducted in Romanian dairy-cattle and pig farms documented high concentrations of airborne microorganisms within livestock facilities and, in the case of pig farms, microbial contamination in outdoor air surrounding production units [104,105]. Airborne microbial communities have also been characterized in urban particulate matter in north-eastern Romania, further demonstrating that atmospheric microbial exposure extends beyond livestock-production settings, although antimicrobial resistance was not specifically investigated in that study [106]. These findings support bioaerosols as a potential route for movement of animal-associated microorganisms beyond farm buildings. Nevertheless, antimicrobial susceptibility testing, resistance-gene characterization, and genomic comparison of airborne *E. coli* or *Salmonella* with animal isolates were not performed.

Accordingly, landfill and bioaerosol studies should be considered contextual evidence of possible dissemination pathways, rather than evidence that resistant *E. coli* or *Salmonella* are actively transmitted through air in Romania. Future studies combining culture, susceptibility testing, ARG detection, and WGS of contemporaneously collected animal and airborne isolates would be required to establish the importance of this route.

Romanian environmental evidence is strongest for wastewater and aquatic matrices, where antimicrobial residues, resistant *Enterobacterales*, *E. coli*, and several clinically relevant ARGs have been documented. Hospital, municipal, and farm effluents should be considered distinct environmental sources because they differ in origin, selective pressures, and potential routes of exposure. In contrast, landfill and bioaerosol studies currently provide mainly contextual evidence of microbial dispersal. Integrated studies simultaneously sampling livestock, wastewater, manure, soil, surface water, and human-associated sources remain a major priority for determining whether resistant strains or mobile resistance determinants circulate between these compartments.

## 8. Integration Across the One Health Spectrum and Molecular Epidemiology

Across the reviewed interfaces, the key integrative question is not whether resistance occurs in multiple compartments, but what type of evidence links those observations. Phenotypic similarity and shared ARGs provide evidence of overlap; strain typing and genomic relatedness provide progressively stronger evidence of connectivity; direct transmission additionally requires epidemiologically linked and temporally resolved sampling.

Several resistance features recur across sectors, including ampicillin and other β-lactam resistance, *tet*racycline, *sul*fonamide, and fluoroquinolone resistance, together with *bla*TEM, CTX-M-type β-lactamase genes, *bla*CMY-2, *qnr*, *tet*, *sul*, and class 1 integron-associated determinants [29,30,31,33,34,38,39,40,44,46,58,61,83,85,90,91,101]. Their recurrence identifies common mechanisms but does not establish movement of the same strain or mobile element between sources. Table 1 summarizes the distribution, study volume, sample contexts, and molecular depth of the available evidence across the principal One Health interfaces considered in this review.

### 8.1. β-Lactam Resistance: ESBL and AmpC Mechanisms

The molecular evidence is strongest for β-lactam resistance in *E. coli*. ESBL-producing isolates carrying CTX-M-type genes, including CTX-M-15, have been identified in poultry-associated populations, food, companion animals, and human-linked investigations [30,31,33,34,83,85]. AmpC-associated resistance has also been reported in poultry and swine populations [30,42,44,45]. These mechanisms should be distinguished because ESBL and AmpC enzymes differ in their antimicrobial spectrum, epidemiological behavior, and genetic context.

In several studies, *bla*TEM occurred together with other resistance determinants, while CTX-M-type genes were particularly important among extended-spectrum cephalosporin-resistant *E. coli*. However, most Romanian investigations used targeted PCR and did not resolve whether individual ARGs were chromosomal or plasmid-borne, whether multiple determinants were co-located on the same mobile element, or whether identical plasmids occurred across epidemiologically connected hosts. Therefore, the evidence currently supports widespread occurrence of transferable β-lactam-resistance mechanisms but only limited conclusions regarding the vehicles responsible for their dissemination.

The study by Cozma et al. [85] provides a higher level of resolution because WGS was used to characterize extended-spectrum cephalosporin-resistant *Enterobacterales* from dogs and humans sharing households or shelter environments. In addition to sequence-type assignment and resistance-gene characterization, plasmid replicons were investigated. Closely related *E. coli* isolates were identified in animals and humans sharing the same environment, providing evidence compatible with recent cross-host circulation or exposure to a shared source. Even in this setting, transmission direction could not be established from genomic similarity alone.

### 8.2. Quinolone, Tetracycline, Sulfonamide, and Integron-Associated Resistance

Plasmid-mediated quinolone resistance represents another recurrent mechanism. *qnr* determinants have been reported in poultry-, swine-, food-, and companion-animal-associated Enterobacterales [33,40,46,83]. In western Romanian swine populations, *qnr*B and *qnr*S were identified among ciprofloxacin-resistant *E. coli* [46], while Colobatiu et al. [40] identified plasmid-mediated quinolone resistance(PMQR) determinants in *Salmonella* obtained from human, food, and companion-animal sources. These findings support the contribution of mobile quinolone-resistance mechanisms, but the extent of plasmid sharing between sectors remains largely unresolved.

Tetracycline- and sulfonamide-resistance determinants are even more widely distributed. Genes such as *tet*A, *tet*B, *tet*C, *sul*1, *sul*2, and *sul*3 have been identified among livestock-, food-, companion-animal-, and wildlife-associated *E. coli* and *Salmonella* [33,34,38,39,58,61,90,91]. Class 1 integrons have additionally been reported in cattle-associated and environmental isolates [58,61,101]. Because integrons can capture and express resistance-gene cassettes and frequently occur in association with other mobile genetic elements, their detection is epidemiologically relevant. Nevertheless, the available Romanian studies generally do not provide sufficient long-read or plasmid-resolved genomic information to determine whether similar integrons or ARG combinations represent dissemination of the same mobile element across sectors.

### 8.3. Clonal Relatedness Versus Mobile-Element Dissemination

A central interpretive challenge is distinguishing clonal dissemination of resistant bacteria from horizontal transfer of resistance determinants. Similar resistance profiles or ARG combinations can arise because the same bacterial lineage has spread between hosts, because mobile elements have transferred between unrelated bacteria, or because comparable antimicrobial-selection pressures have independently favored similar resistance mechanisms.

Only a small number of Romanian studies provide evidence approaching strain-level source comparison. Maciuca et al. [31] identified CTX-M-15-producing *E. coli* among poultry and human isolates, demonstrating overlap in clinically relevant resistance mechanisms across sectors, but this does not by itself prove direct poultry-to-human transmission. Colobatiu et al. [40] found PFGE similarity among *S. Infantis* isolates recovered from a dog, chicken meat, and a human source, providing stronger cross-source molecular evidence, although PFGE has lower discriminatory power than WGS and the isolates were not collected within a prospectively linked epidemiological investigation.

The strongest genomic evidence currently comes from the dog–human study of Cozma et al. [85], where WGS demonstrated closely related resistant *E. coli* within shared environments. In the aquatic environment, Farkas et al. [101] also identified identical ERIC-PCR fingerprints among selected *Enterobacterales* from different water-associated matrices, suggesting environmental connectivity. However, these studies address specific interfaces rather than an integrated animal–food–environment continuum.

The clinically important *E. coli* ST131 lineage has also been reported in Romanian environmental literature [98]. However, the available evidence does not provide epidemiologically linked genomic comparison between environmental ST131 isolates and corresponding animal, food, or human isolates. Its detection should therefore be interpreted as evidence of environmental occurrence of a clinically relevant lineage rather than as demonstration of cross-sector transmission.

Clinically important lineages deserve particular attention when high-resolution typing data are available. Sequence-type information has been reported in selected Romanian investigations, but lineage-level evidence remains much less extensive than targeted ARG data. Accordingly, future studies should combine MLST or cgMLST/SNP analysis with plasmid reconstruction, ARG localization, and epidemiological metadata to distinguish dissemination of successful clones from dissemination of mobile resistance elements.

Overall, the strength of evidence supporting cross-sector connections varies substantially among Romanian studies. The detection of resistant *E. coli* or *Salmonella* spp. in multiple compartments demonstrates co-occurrence, while the identification of similar resistance phenotypes or ARGs indicates overlap but does not establish epidemiological connectivity. Molecular typing, such as PFGE or Enterobacterial Repetitive Intergenic Consensus (ERIC) PCR, provides stronger evidence of relatedness, whereas WGS offers higher-resolution assessment of potential clonal circulation. Even when closely related isolates are identified, however, direct transmission and its direction cannot be inferred without epidemiologically linked and temporally resolved sampling. The current evidence therefore supports cross-sector AMR connections with different levels of molecular support rather than a single demonstrated transmission pathway.

Future One Health investigations should therefore integrate contemporaneous sampling, WGS, plasmid-resolved analysis, and detailed epidemiological metadata to distinguish shared exposure, clonal dissemination, horizontal gene transfer, and direct transmission.

## 9. Knowledge Gaps

Despite the growing Romanian literature on antimicrobial-resistant *Escherichia coli* and *Salmonella* spp., several gaps continue to restrict comparison between reservoirs and interpretation within a One Health framework. The available evidence is both methodologically heterogeneous and geographically uneven, with many investigations conducted in restricted regions rather than through nationally representative sampling. Studies also differ considerably in sampled populations, clinical status, sample matrices, antimicrobial panels, susceptibility-testing methods, interpretive criteria, and definitions of multidrug resistance. As a result, resistance percentages reported by individual studies cannot be interpreted as directly comparable national prevalence estimates. Greater methodological harmonization and more complete reporting of sampling and laboratory procedures would substantially improve future cross-study comparisons.

A second major gap is the scarcity of epidemiologically linked longitudinal investigations. Most Romanian studies examine individual compartments independently for example livestock, slaughter samples, food products, companion animals, or wastewater rather than following isolates across successive stages of a production or exposure pathway. Consequently, the detection of similar resistance phenotypes, serovars, or ARGs in different compartments generally demonstrates overlap rather than transmission. Studies combining contemporaneous sampling of animals, food, humans, and relevant environmental matrices are needed to determine whether resistant strains persist across these interfaces.

The evidence base is also uneven among animal species and food matrices. Poultry and swine are comparatively well represented, whereas cattle-associated *Salmonella* remains particularly underexplored. No eligible Romanian primary study providing antimicrobial-susceptibility data for *Salmonella* from cattle, calves, bovine feces, bovine carcasses, raw beef, or cattle-associated farm environments was identified in the evidence evaluated for this review. Food studies are similarly concentrated on poultry meat and pork. Evidence from milk and dairy products has increased, including data from raw cow milk, raw ovine milk, and raw-milk cheeses, whereas AMR evidence from eggs remains based on a small number of isolates. These imbalances limit assessment of the relative importance of different food-animal pathways.

Environmental surveillance represents another important gap. Romanian studies provide direct evidence of antimicrobial residues, resistant *Enterobacterales*, *E. coli*, and ARGs in hospital, municipal, farm, and aquatic environments, but these investigations remain methodologically heterogeneous and are rarely linked to corresponding animal, food, or human isolates. Evidence from manure, agricultural soils, and livestock-associated runoff is particularly sparse at the primary-study level. Landfill and bioaerosol studies currently demonstrate microbial dissemination rather than organism-specific AMR transmission and should therefore be regarded as contextual evidence. Coordinated animal–effluent–soil–water sampling would be required to clarify the environmental persistence and redistribution of resistant bacteria and mobile resistance determinants.

A further limitation concerns the depth of molecular and genomic characterization. Targeted PCR has demonstrated numerous clinically relevant determinants, including CTX-M-type β-lactamases, *bla*TEM, AmpC-associated genes, *qnr*, *tet*, *sul*, and integron-associated resistance determinants. However, relatively few studies resolve whether these genes are chromosomal or plasmid-borne, whether multiple ARGs are co-localized on the same mobile element, or whether similar resistance patterns reflect clonal dissemination rather than horizontal gene transfer. Whole-genome sequencing remains uncommon across epidemiologically connected Romanian reservoirs, although the dog–human study provides an important example of the additional resolution that genomic analysis can offer. Broader use of WGS, cgMLST or SNP-based analysis, and plasmid-resolved sequencing would strengthen source attribution and molecular epidemiology.

Human evidence also remains incompletely integrated with the animal–food–environment literature. Several Romanian investigations have compared human isolates with those from poultry, swine, food, or companion animals, but these studies represent selected interfaces rather than a systematic synthesis of human clinical *E. coli* and *Salmonella* AMR. This reflects the scope of the present review, which focuses primarily on animal–food–environment interfaces, but it also highlights the need for surveillance systems capable of linking veterinary, food-safety, environmental, and human-health data using compatible metadata and analytical approaches.

Finally, the major challenge is cross-sector integration. Romanian AMR information is generated through research studies, national and European surveillance activities, clinical laboratories, veterinary monitoring, food-safety programs, and environmental investigations, but these data streams differ in structure and purpose. More interoperable systems linking epidemiological metadata, antimicrobial susceptibility results, antimicrobial-use information, resistance determinants, and genomic data would allow more robust comparison between sectors and facilitate early recognition of emerging resistance patterns.

The uneven distribution of the Romanian evidence base is summarized in Figure 2, which maps the availability of phenotypic, molecular, genomic, and epidemiologically linked evidence across the principal One Health interfaces considered in this review.

The principal gaps in the Romanian evidence base therefore concern methodological comparability, epidemiologically linked sampling, underrepresented reservoirs such as cattle-associated *Salmonella* and selected food matrices, environmental integration, and high-resolution genomic epidemiology. Addressing these gaps would shift Romanian AMR research from largely parallel compartment-specific studies toward a more integrated One Health framework capable of distinguishing shared resistance patterns from actual cross-sector circulation.

## 10. Policy, Surveillance, and Future Directions

Romania has established a national policy framework for AMR control through the National Strategy for the Prevention and Limitation of Healthcare-Associated Infections and Combating Antimicrobial Resistance for 2023–2030 [107]. This framework is complemented by participation in harmonized European monitoring of *Salmonella* spp., commensal *E. coli*, and ESBL-/AmpC-/carbapenemase-producing *E. coli* under Commission Implementing Decision (EU) 2020/1729 [16]. Together, these mechanisms provide an important foundation for One Health AMR surveillance, although the evidence synthesized in this review indicates that stronger integration is still needed across veterinary, food, environmental, and relevant human-health data streams.

A first priority is continued strengthening of antimicrobial stewardship in food-producing animals. Romanian veterinarians have reported variable use of susceptibility testing, broad-spectrum antimicrobial use, prophylactic administration, and antimicrobial combinations [14]. These findings should, however, be interpreted in the context of current legislation. Regulation (EU) 2019/6, applicable since January 2022, prohibits routine antimicrobial use and restricts prophylactic use to exceptional circumstances; for antibiotics, prophylaxis is restricted to individual animals [15]. Metaphylactic use is permitted only when the risk of spread within a group is high and appropriate alternatives are unavailable. Consequently, future stewardship efforts should focus not only on reducing overall exposure but also on appropriate diagnosis, greater use of antimicrobial susceptibility testing where clinically relevant, compliance with current prescribing restrictions, and improved farm-level recording of antimicrobial use.

Monitoring antimicrobial consumption should also become more closely linked to resistance surveillance. Although veterinary antimicrobial sales in Romania have declined in recent years [108], sales data alone cannot identify the animal species, production stage, disease indication, or farm-level practices most strongly associated with resistance selection. Species-level and, where feasible, farm-level antimicrobial-use data would allow more informative comparison with AMR patterns in poultry, swine, cattle, and other food-producing animals. This is consistent with broader European objectives to reduce unnecessary antimicrobial exposure and with the EU target of reducing overall sales of antimicrobials for farmed animals and aquaculture by 50% by 2030 [109].

A second priority is the development of more integrated surveillance across One Health compartments. Current Romanian monitoring provides important animal, food, and human-health data, but the systems remain largely compartmentalized [7,9]. Future surveillance would benefit from coordinated protocols linking livestock, slaughter and processing environments, foods, companion animals where epidemiologically relevant, wastewater, surface water, and selected human populations. This approach is consistent with WHO guidance promoting integrated One Health surveillance of ESBL-producing *E. coli* across human, animal, food, and environmental compartments [110]. Such integration does not require all sectors to collect identical data, but core variables, including sampling date, geographic area, source, host status, antimicrobial susceptibility method, interpretive standard, and molecular characteristics, should be sufficiently standardized to permit meaningful comparison.

Environmental surveillance should be incorporated more systematically into this framework. Romanian studies have documented resistant *Enterobacterales*, ARGs, and antimicrobial residues in hospital, municipal, farm, and aquatic environments [99,100,101], yet environmental sampling remains less standardized than monitoring in food-producing animals. Wastewater is a particularly suitable component for expansion because it can integrate signals from healthcare, community, and agricultural sources. Recent European environmental policy work has likewise emphasized stronger monitoring of AMR in surface waters and greater integration of environmental information into AMR risk management [111]. Agricultural pathways, including manure, farm effluent, runoff, and receiving waters, should also receive greater attention where they can be epidemiologically linked to animal-production systems.

A third priority is the expansion of genomic epidemiology. Targeted PCR has provided valuable information on resistance determinants, but it cannot reliably establish whether similar genes in different reservoirs are located on the same mobile element or carried by closely related bacterial lineages. Wider use of WGS would allow sequence-type assignment, resistance-gene characterization, plasmid and mobile-element analysis, and high-resolution assessment of isolate relatedness. The integration of WGS into AMR surveillance is also supported by WHO GLASS guidance, which highlights genomic approaches as complementary tools for characterization, surveillance, and investigation of resistant bacterial populations [112]. Existing initiatives such as PANDOMIC, which has supported genomic surveillance capacity and Salmonella sequencing in Romania [113], provide a foundation on which broader One Health genomic surveillance could be developed. Genomic surveillance should be prioritized particularly for epidemiologically connected interfaces, such as farm–slaughter–food chains, animal–human shared environments, and linked wastewater or surface-water systems. Where possible, genomic data should be interpreted together with temporal, spatial, and exposure information rather than being used alone to infer transmission.

Improved data interoperability is equally important. AMR findings generated through veterinary surveillance, food-safety programs, clinical laboratories, environmental investigations, and research projects are currently collected for different purposes and frequently use different metadata structures. A national One Health AMR framework capable of linking phenotypic susceptibility data, antimicrobial-use information, resistance determinants, genomic data, and epidemiological metadata would facilitate recognition of emerging patterns and enable more robust source attribution. Such integration also requires appropriate governance structures, including clearly defined responsibilities, cross-sector coordination, data-sharing mechanisms, and sustained collaboration among participating institutions [114]. European policy increasingly emphasizes this integrated approach, including stronger national One Health action plans, surveillance of both AMR and antimicrobial consumption, and greater environmental inclusion [9,111,114].

Finally, surveillance expansion should remain risk-based. The gaps identified in this review suggest that priority should be given to interfaces where evidence is currently weakest or where clinically important resistance could have substantial public-health implications. These include cattle-associated *Salmonella*, underrepresented food matrices, environmental pathways linked to livestock production, and cross-sector investigations capable of distinguishing shared resistance mechanisms from actual strain circulation. At the same time, well-characterized sectors such as poultry and swine should continue to be monitored because they provide the strongest longitudinal basis for detecting changes in resistance trends.

Future Romanian AMR policy should build on existing European surveillance and veterinary-medicinal-product legislation while strengthening antimicrobial stewardship, species-level antimicrobial-use monitoring, environmental surveillance, genomic epidemiology, and interoperability of human, animal, food, and environmental data. The objective should not simply be to generate more AMR data, but to produce sufficiently standardized and epidemiologically connected information to determine where resistant *E. coli* and *Salmonella* occur, how resistance determinants are maintained, and which cross-sector connections are supported by robust evidence.

## 11. Conclusions

Antimicrobial-resistant *Escherichia coli* and *Salmonella* spp. are documented across multiple Romanian animal, food, human-associated, and environmental compartments, with the most substantial evidence derived from poultry, swine, meat products, and selected companion-animal and wastewater studies. Recurrent resistance involves β-lactams, tetracyclines, sulfonamides, and fluoroquinolones, while commonly reported determinants include CTX-M-type genes, *bla*TEM, *qnr*, *tet*, *sul*, and integron-associated markers. However, the available studies are heterogeneous in design, sampling, susceptibility-testing methods, and molecular resolution, which limits direct comparison and prevents interpretation of individual study estimates as national prevalence values.

The evidence supports overlapping AMR patterns across One Health compartments, while evidence compatible with recent cross-host or cross-source circulation is limited to selected settings and generally does not establish transmission direction. Important gaps remain in epidemiologically linked sampling, cattle-associated *Salmonella*, environmental integration, and high-resolution genomic characterization across connected animal–food–human–environment interfaces.

Future progress should focus on harmonized surveillance, stronger antimicrobial stewardship, improved integration of environmental monitoring, and broader use of whole-genome and plasmid-resolved approaches together with detailed epidemiological metadata. Such developments would allow Romanian AMR surveillance to move from compartment-specific description toward more robust identification of shared sources, transmission pathways, and emerging resistance threats.

## Figures and Tables

**Figure 1:**
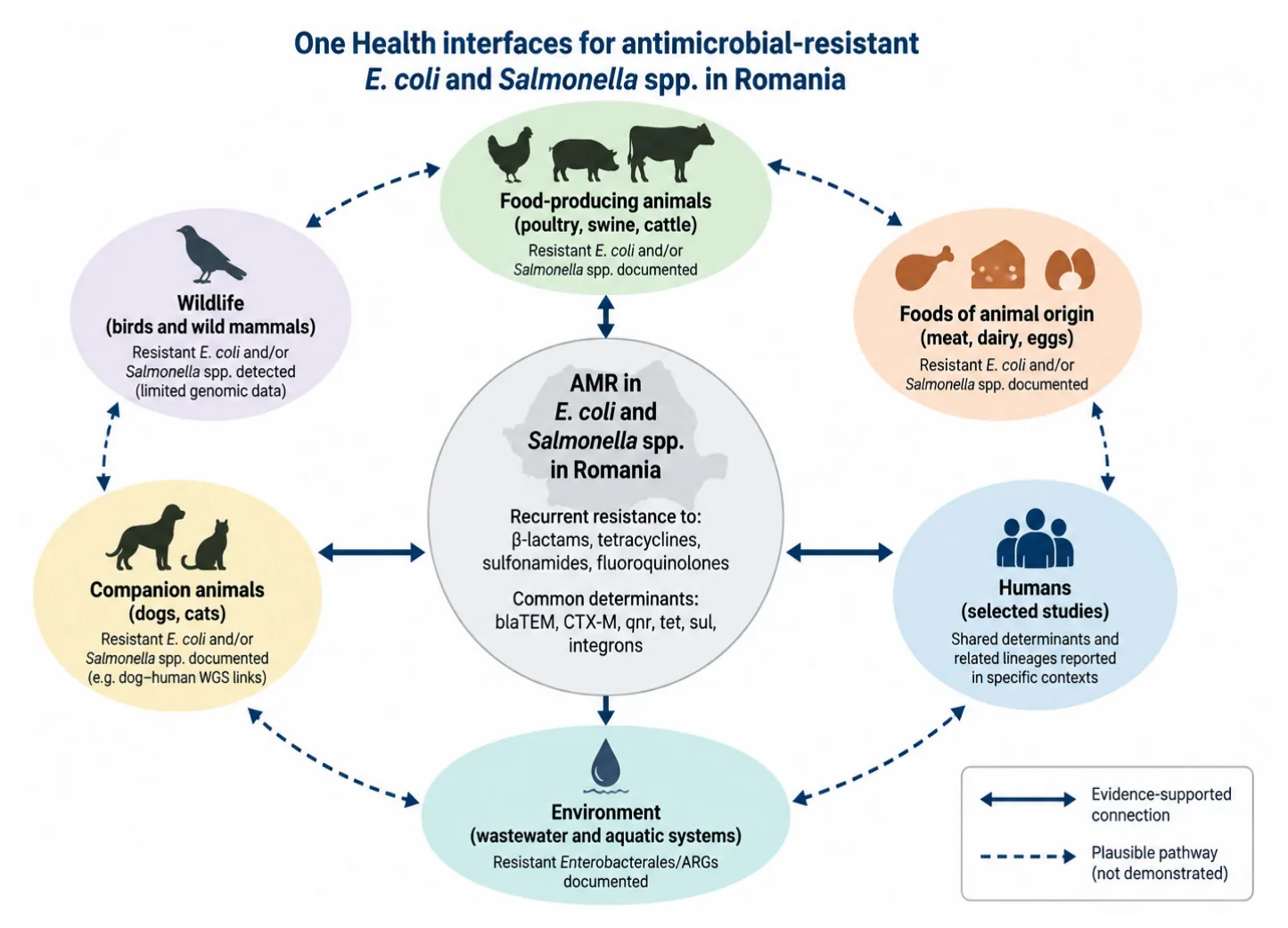
One Health interfaces for antimicrobial-resistant *Escherichia coli* and *Salmonella* spp. in Romania. The figure summarizes the main animal, food, human-associated, and environmental compartments represented in Romanian studies. Solid arrows indicate evidence-supported connections, whereas dashed arrows indicate plausible pathways for which direct transmission has not been demonstrated.

**Figure 2:**
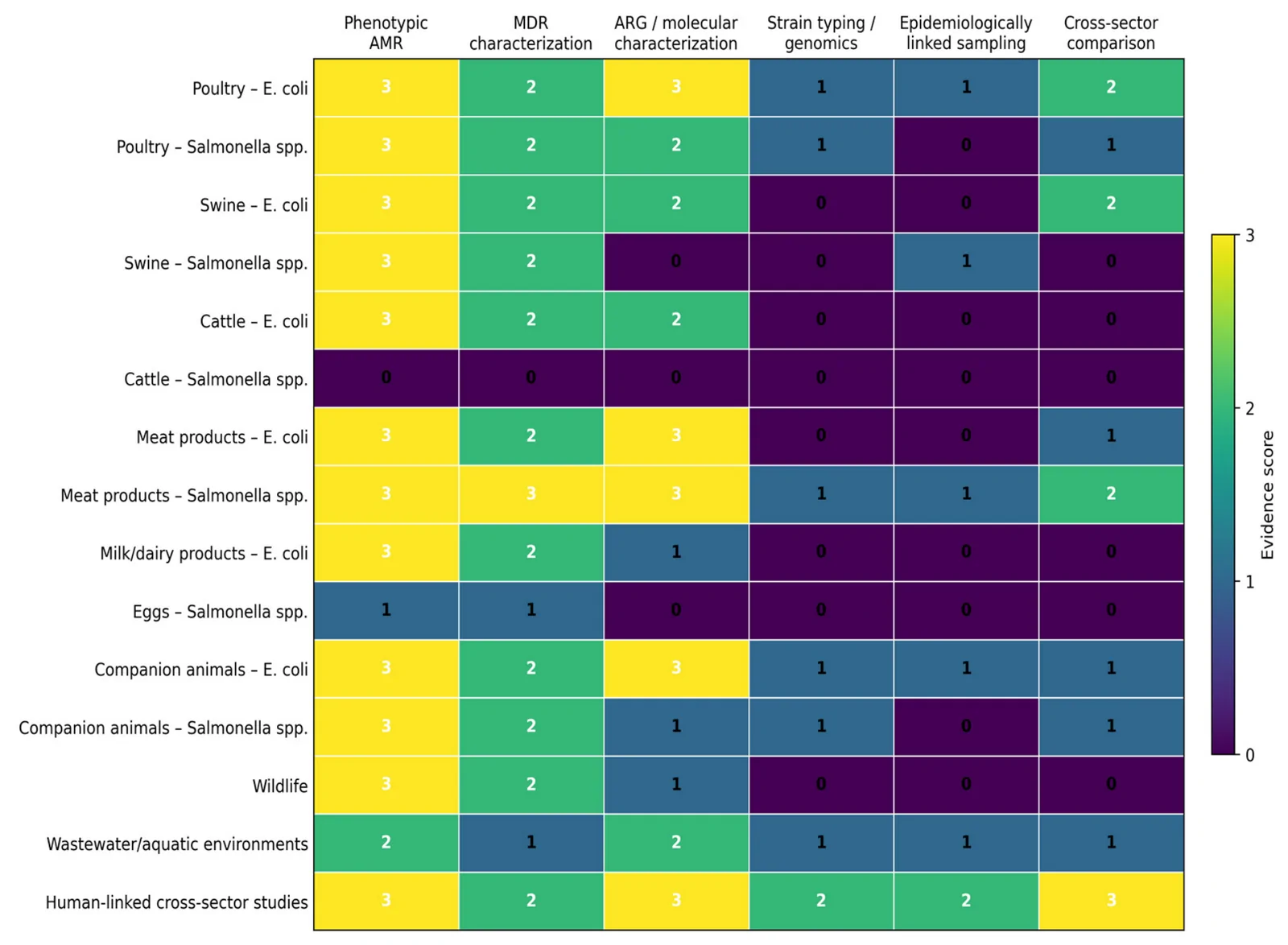
Evidence coverage of Romanian antimicrobial-resistance research across major One Health interfaces. Scores represent the number of eligible primary studies providing each type of evidence: 0, no study; 1, one study; 2, two to three studies; and 3, four or more studies. The heatmap represents evidence coverage rather than AMR prevalence or resistance severity.

**Table 1:** Summary of evidence on antimicrobial resistance in Escherichia coli and Salmonella spp. across major One Health interfaces.

Reservoir/ Interface	Target Organism	Romanian Studies, *n*	Publication Years	Main Populations/Matrices	Samples/Isolates Represented *	Molecular Characterization Reported	WGS/Epidemiologically Linked Evidence	References
Poultry	*E. coli*	8	2014–2026	Cecal/intestinal samples from slaughter birds, broilers and turkeys; carcasses; chicken meat; slaughter-associated samples	Study sizes ranged from relatively small isolate collections to national monitoring datasets; examples include 100 samples/30 isolates [33], 444 samples/201 isolates [28], 840 commensal isolates [32]	ESBL/AmpC characterization; *bla*TEM, CTX-M-type genes, *bla*CMY-2, PMQR, *tet*, *sul* and other determinants	Molecular comparisons between poultry and human isolates reported [31], but no epidemiologically linked poultry–slaughter–retail WGS study represented	[28,29,30,31,32,33,34,39]
	*Salmonella* spp.	6	2014–2022	Poultry carcasses, raw poultry meat and meat products, food-chain samples	Examples include 42 isolates [36] and 112 isolates [38]; some studies included poultry together with other food matrices	Serotyping; β-lactamase, PMQR, *tet*, *sul*, *dfr* and other determinants in selected studies	Cross-source PFGE evidence reported [40], but no epidemiologically linked poultry-chain WGS study represented	[35,36,37,38,39,40]
Swine	*E. coli*	6	2017–2025	Clinically healthy slaughter pigs, caecal samples, grower pigs/piglets, farm fecal samples	Examples include 93 pig isolates [29], 223 ESBL/AmpC-producing isolates [42], 76 isolates [44], and 45 ESBL-producing isolates [45]	ESBL/AmpC phenotypes; *bla*TEM, *amp*C, *bla*Z, *qnr*B/*qnr*S, *tet*K and other determinants	Human–swine molecular comparisons reported [46]; no linked farm–slaughter–pork WGS investigation represented	[29,42,43,44,45,46]
	*Salmonella* spp.	6	2014–2024	Slaughter-process samples, pork, retail products and traditional slaughter products	Examples include 26 isolates [41] and 48 pork-derived isolates [48]; other studies combined several food matrices	Serotyping and selected molecular characterization; ARG characterization less extensive than for swine *E. coli*	No epidemiologically linked swine–slaughter–food WGS study represented	[35,37,41,43,47,48]
Cattle/dairy cattle	*E. coli*	4	2017–2025	Slaughter-associated/pathogenic intestinal isolates, dairy-cattle mastitis, postpartum uterine infections, and young cattle/calves	Examples include 29 pathogenic Romanian slaughter-cattle isolates [58] and 16 mastitis-associated *E. coli* isolates [60]; ref. [61] contains a mixed young-animal collection including calves	*tet*, *sul*, aminoglycoside and trimethoprim resistance genes; class 1 integrons reported in selected studies	No epidemiologically linked cattle–milk–food WGS study represented	[58,59,60,61]
	*Salmonella* spp.	0	—	—	—	—	—	—
Foods of animal origin–poultry/pork and other meats	*E. coli*	6	2014–2026	Chicken carcasses/meat, pork/minced meat, meat packaging and other meat products	Study-level denominators vary substantially	ESBL-associated resistance and selected resistance-gene characterization	No comprehensive genomic tracing from animal source through slaughter/processing to final retail product represented	[28,33,34,39,62,63]
	*Salmonella* spp.	9	2014–2024	Poultry meat, pork, sausages, processed meat and other retail products	Examples include 51 food-derived isolates [47] and 48 pork isolates [48]; larger monitoring studies included multiple matrices	Serotyping and selected β-lactamase/PMQR/other resistance determinants	Cross-source PFGE reported [40]; limited genomic food-chain linkage	[35,36,37,38,39,40,47,48,62]
Milk and dairy products	*E. coli*	4	2014–2025	Raw cow milk, raw ovine bulk-tank milk, raw-milk cheese, milk and other dairy products	53 cheese samples/43 isolates [75]; 1,696 raw cow-milk samples [73]; 72 raw ovine-milk samples/48 isolates [74]	Mainly phenotypic AMR; selected virulence characterization; *stx2* investigated in raw-milk studies	No WGS-based farm–milk–dairy-product tracing represented	[62,73,74,75]
Eggs	*Salmonella* spp.	1	2020	Shell eggs sampled together with other foods of animal origin	48 egg samples; 3 *Salmonella* isolates	Serotyping: *S. Enteritidis* and *S. Infantis*; phenotypic AMR	No genomic linkage	[47]
Companion animals	*E. coli*	7	2022–2026	Healthy dogs, dogs with otitis, household/shelter dogs, reptiles, pet birds, rabbit and guinea pig	Examples include 124 healthy-dog samples [83], 54 canine *E. coli* isolates [84], and 263 dog/human samples [85]; some evidence derives from individual case reports	ESBL and PMQR determinants; *bla*CTX-M, *bla*TEM, *tet*A, *sul*1 and other ARGs	Illumina WGS demonstrated closely related ESC-resistant *E. coli* between dogs and humans sharing environments [85]	[83,84,85,87,89,90,91]
	*Salmonella* spp.	3	2015–2023	Urban cats, pet reptiles and one cross-source canine isolate	16 isolates from 85 cats [86]; 29 reptile-level isolates analyzed for MDR [88]; one dog isolate in the cross-source study [40]	Serotyping; PMQR and β-lactamase determinants in [40]	PFGE showed cross-source relatedness among dog, food and human *S. Infantis* isolates [40], but this does not establish transmission	[40,86,88]
Wildlife	*E. coli*/*Salmonella* spp.	3	2023–2026	Fallow deer nasal/rectal samples, red fox samples, mesenteric lymph nodes from wild boars and cervids	240 fallow-deer samples [93]; 216 red-fox samples with 56 *E. coli* and 2 *S. enterica* isolates [94]; 103 wild ungulates with 75 *E. coli* isolates [95]	Predominantly phenotypic AMR characterization	No epidemiologically linked wildlife–livestock–human WGS investigation represented	[93,94,95]
Wastewater and aquatic environments	Primarily *E. coli*/Enterobacterales; broader ARG evidence	3	2017–2025	Hospital wastewater, municipal WWTP influent/effluent, surface water, groundwater and farm effluent	Ref. [101] characterized 678 Enterobacterales isolates from multiple aquatic matrices, including resistant *E. coli*; ref. [100] included seasonal WWTP sampling and phenotypic testing	*bla*TEM-1, *tet*A, *intI*1, *sul*1, *qacEΔ1* and other ARGs; ERIC-PCR in [101]	Molecular similarity across some aquatic matrices reported, but no integrated animal–food–environment WGS investigation	[99,100,101]
Landfill and livestock bioaerosol pathways	Contextual microbial evidence	3	2008–2022	Landfill air, dairy-cattle farm air, pig-farm indoor/outdoor air	Organism-specific AMR isolate totals not applicable	No target-organism ARG characterization	No genomic evidence; these studies support potential microbial dissemination only	[103,104,105]
Human-linked cross-sector evidence	*E. coli*/ *Salmonella* spp.	5	2015–2025	Human isolates compared with poultry, livestock, food, hospital/swine or companion-animal sources	Sample sizes differ by study and are reported individually in	ESBL, PMQR and other shared resistance determinants characterized across selected compartments	WGS-supported dog–human relatedness [85]; PFGE and other molecular comparisons in [31,40,46] provide lower-resolution evidence	[29,31,40,46,85]

Note: * Study counts are interface-specific and are not additive because individual publications may contribute evidence to more than one reservoir or matrix. Sample and isolate numbers are reported as presented in the original studies and should not be interpreted as pooled prevalence denominators. Contextual studies without organism-specific AMR characterization are identified separately.

## Data Availability

No new data were created or analyzed in this study.
